# Combining large language models with enterprise knowledge graphs: a perspective on enhanced natural language understanding

**DOI:** 10.3389/frai.2024.1460065

**Published:** 2024-08-27

**Authors:** Luca Mariotti, Veronica Guidetti, Federica Mandreoli, Andrea Belli, Paolo Lombardi

**Affiliations:** ^1^Department of Physics, Informatics and Mathematics, Università di Modena e Reggio Emilia, Modena, Italy; ^2^Expert.ai, Modena, Italy

**Keywords:** LLMS, knowledge graph, relation extraction, knowledge graph enrichment, AI, enterprise AI, carbon footprint, human in the loop

## Abstract

Knowledge Graphs (KGs) have revolutionized knowledge representation, enabling a graph-structured framework where entities and their interrelations are systematically organized. Since their inception, KGs have significantly enhanced various knowledge-aware applications, including recommendation systems and question-answering systems. Sensigrafo, an enterprise KG developed by Expert.AI, exemplifies this advancement by focusing on Natural Language Understanding through a machine-oriented lexicon representation. Despite the progress, maintaining and enriching KGs remains a challenge, often requiring manual efforts. Recent developments in Large Language Models (LLMs) offer promising solutions for KG enrichment (KGE) by leveraging their ability to understand natural language. In this article, we discuss the state-of-the-art LLM-based techniques for KGE and show the challenges associated with automating and deploying these processes in an industrial setup. We then propose our perspective on overcoming problems associated with data quality and scarcity, economic viability, privacy issues, language evolution, and the need to automate the KGE process while maintaining high accuracy.

## 1 Introduction

A Knowledge Graph (KG) represents real-world knowledge using a graph structure, where nodes denote entities and edges represent relationships between them (Hogan et al., 2021). Since Google introduced the Knowledge Graph in 2012, KGs have become essential in knowledge representation, enhancing various tasks Companies use them to improve product performance, boosting data representation and transparency in recommendation systems, efficiency in question-answering systems, and accuracy in information retrieval systems (Peng et al., 2023).

This work presents the perspective of Expert.AI, a leading enterprise in Natural Language Understanding (NLU) solutions, centered on meticulously created and curated KGs by expert linguists. While manual curation ensures high precision and data quality, it demands significant human effort, and the rapid evolution of real-world knowledge requires frequent updates to KGs.

Recent advancements in Large Language Models (LLMs) suggest potential for partial automation of this process. LLMs, deep learning architectures designed for natural language processing, have demonstrated impressive results in NLU tasks. Their advanced capabilities represent a promising avenue for automating and enhancing Knowledge Graph Enrichment (KGE), refining, and adding new entities and relationships in KGs. By leveraging the implicit knowledge embedded within pre-trained LLMs (PLMs), companies can streamline the identification of new entities and relationships in external corpora, enriching their KGs with minimal manual intervention (Valmeekam et al., 2024).

However, automating KGE from external text in an industrial context is far from straightforward. It is crucial to choose an appropriate methodological framework: various PLM-based KGE techniques require model finetuning, while others rely on prompting. We will discuss the advantages and disadvantages of both approaches. For instance, while finetuning is generally costly and requires large amounts of annotated data, prompting is more cost-effective but poses privacy-related risks.

We will also examine the primary challenges of implementing corporate KGE solutions, categorizing them into four areas: (i) the quality and quantity of public or automatically annotated data, (ii) developing sustainable solutions in terms of computational resources and longevity, (iii) adaptability of PLM-based KGE systems to evolving language and knowledge, and (iv) creating models capable of efficiently learning the KG structure.

We review existing solutions for each issue and identify promising options for automating KGE in industrial settings using PLMs while maintaining high quality. We recommend a hybrid approach that combines PLMs, KG structure understanding, and domain expertise, ensuring privacy compliance. To adapt to evolving LLMs, we suggest treating PLMs as plug-and-play components for versatility and longevity.

This paper is structured as follows: Section 2 presents Expert.AI and its research investment objectives. Section 3 discusses the state-of-the-art in PLM-based KGE. Section 4 provides our perspective on the challenges of deploying these methods in an enterprise environment. Finally, conclusions are drawn in Section 5.

## 2 Sensigrafo: an enterprise KG and its characteristics

Expert.AI, formerly known as Expert System, is a leading AI enterprise specializing in solving complex language challenges. With over 300 natural language solutions, Expert.AI has transformed language-intensive processes across various sectors. Central to Expert.AI's NLU solutions is a collection of large KGs called *Sensigrafo*s, meticulously built by linguists and domain experts and carefully modified to gain performance in downstream NLU tasks. ^1^ Each Sensigrafo includes attributes like grammatical role, semantic relation, definition/meaning, domain, and frequency that establish the characteristics of words and concepts (Buzzega et al., 2023). Terms with the same meaning are grouped into syncons, interconnected by millions of logical and linguistic links, organized hierarchically. For example, the English Sensigrafo contains about 440,000 syncons, grouping more than 580,000 words, and 80+ relation types, yielding around 7.1 million links.

In contrast, most collaborative open-source KGs are generated automatically, resulting in numerous triples. DBpedia, for instance, contains about 900 million triples. The number of entity classes varies across KGs, with Wikidata having over 110 million items and 500 million facts, and YAGO encompassing knowledge of more than 67 million entities and 343 million facts (Suchanek et al., 2023). The number of relation types also varies, with Freebase having 1,345 and YAGO holding only 140 (Suchanek et al., 2023). These KGs span diverse domains, primarily derived from text corpora like Wikipedia, aiming to cover extensive real-world knowledge. Conversely, each Sensigrafo is carefully constructed using only information sources from its intended domain, making the information extraction operation much more reliable and accurate.

However, the accuracy of Sensigrafo's information comes at a high maintenance cost. Adding new syncons and relations requires full human supervision, aided by Expert.AI's semantic engine, Cogito. Cogito uses a Sensigrafo to resolve ambiguities related to word meanings and can expand its knowledge through expert input or analyzing tagged content using ML algorithms.

As real-world information grows and the cost of upgrading Sensigrafo increases, Expert.AI plans to integrate symbolic and statistical technologies, combining expert-validated rules with AI methods to automate Sensigrafo updates. This hybrid approach is expected to reduce the costs of developing and maintaining symbolic AI solutions. Nevertheless, any AI solution should be accompanied by a high degree of explainability, robustness, and precision to make enrichment systems transparent and reliable.

To identify the crucial aspects in developing such a solution, we will present state-of-the-art KGE techniques based on PLMs.

## 3 Pretrained LLM for KG management and enrichment

Relation extraction (RE) and named entity recognition (NER) are key challenges in automatic KGE. RE identifies and categorizes relationships between entities in unstructured text, expanding the KG's structure. NER focuses on recognizing, classifying, and linking entities in the text to the knowledge base. These processes are crucial for accurately identifying entities and their interconnections, enhancing KGs. Recent literature highlights two approaches to NER and RE: creating large training sets with hand-curated or extensive automatic annotations to fine-tune LLMs, or using precise natural language instructions, replacing domain knowledge with prompt engineering efforts (Levy et al., 2017; Li et al., 2019; Soares et al., 2019; Peng et al., 2020; Agrawal et al., 2022; Wang et al., 2023).

Supervised methods for NER and RE usually include a pretraining stage followed by zero-shot learning (Wang et al., 2022) or the use of specialized architectures and training setups (Yu et al., 2020; Li et al., 2022b). Due to the lack of large annotated corpora, many approaches for RE and NER rely on distant supervision (DS), an automated data labeling technique that aligns knowledge bases with raw corpora to produce annotated data.

Early DS approaches to RE use supervised methods to align positive and negative pair relations for pre-training language models, followed by few-shot learning to extract relations (Soares et al., 2019; Peng et al., 2020). DS methods for NER involve tagging text corpora with external knowledge sources like dictionaries, knowledge bases, or KGs. A common DS method for NER is the teacher-student framework. For example, Liang et al. (2020) proposed a two-stage method: fine-tuning a LLM on DS labels, followed by teacher-student system self-training with pseudo soft labels to improve performance.

While DS is useful when labeled data is scarce or expensive, it can introduce incomplete and inaccurate labels. To address this, recent works have focused on mitigating DS label noise and improving results (Wan et al., 2022). A common method to address DS noise in RE is multi-instance learning (MIL) (Zeng et al., 2015), which groups sentences into bags labeled as positive or negative with respect to a relation, shifting the RE task from single sentences to bags. However, MIL is not data-efficient, leading to recent extensions into contrastive learning setups. These efforts aim to cluster sentences with the same relational triples and separate those with different triples in the semantic embedding space (Chen et al., 2021; Li et al., 2022a).

Recent years have seen a significant increase in work on NER and RE involving prompt engineering. Prompting for NER includes using entity definitions, questions, sentences, and output examples to guide LLMs in understanding entity types and extracting answers (Ashok and Lipton, 2023; Kholodna et al., 2024). For RE, tasks are rephrased as question-answering (Levy et al., 2017), often injecting latent knowledge contained in relation labels into prompt construction (Chen et al., 2022) and iteratively fine-tuning prompts to enhance the model's ability to focus on semantic cues (Son et al., 2022). In general, zero-shot learning methods have been shown to perform better than supervised settings when the amount of training data is scarce.

Choosing between prompt engineering and fine-tuning is challenging. While prompting with large LLMs like GPTs is appealing for handling complex tasks with minimal data annotation, it can underperform in NER compared to fine-tuned smaller PLMs like BERT derivations, especially with more training data (Gutierrez et al., 2022; Keloth et al., 2024; Pecher et al., 2024; Törnberg, 2024). Large LLMs, such as GPT-3, struggle with specific information extraction tasks, including managing sentences without named entities or relations (Gutierrez et al., 2022). Prompting also faces hallucination issues, often overconfidently labeling negative inputs as entities or relations. Some approaches, such as Wang et al. (2023), address this by enriching prompts and reducing hallucinations via self-verification strategies. Other methods correct inaccurate NER and RE prompting results through active learning techniques (Wu et al., 2022) or by distilling large PLMs into smaller models for specific tasks (Agrawal et al., 2022).

## 4 Perspective

Summarizing the previous sections, the main challenges for enterprise LLM-based solutions for KGE include:

- *Computational resources and longevity:* creating tailored PLM-based KGE solutions can be costly and resource-intensive. There is a need for lightweight, sustainable, and durable training pipelines.
- *Data quality and benchmarking:* collaborative and Enterprise KGs have different structures, causing a mismatch between public benchmark datasets and enterprise use cases.
- *Evolving knowledge:* robust methods are needed to combine automated novelty detection (new links and nodes for the KG) with high-quality human-curated interventions.
- *Lack of adaptive hidden representations:* the learning paradigm should shift from classification to representation learning to accommodate novelty and efficiently encode KG features.

In the following sections, we will provide a comprehensive analysis of each of these challenges.

### 4.1 Computational resources and longevity of solutions

When developing enterprise-level NLU solutions, it's crucial to consider computational resources and carbon footprint due to the high environmental and economic costs of traditional model training (Patil and Gudivada, 2024). Fully fine-tuning PLMs, while effective for specific tasks, is often costly and inefficient, requiring substantial computational resources and time. These models are tailored for narrow applications, making updates challenging (Razuvayevskaya et al., 2023).

In contrast, in-context learning provides greater flexibility, facilitating adaptation to the rapidly evolving field of LLMs. However, prompt engineering is time-consuming and requires methods not universally applicable across models (Zhao et al., 2024). Balancing these factors is essential for creating sustainable and effective NLU solutions that meet the dynamic requirements of modern enterprises (Faiz et al., 2023).

Given the continuous release of new LLMs, we advocate for PLM-based KGE approaches that treat the LLM as a modular component, easily replaceable to integrate context-specific models trained on domain-specific knowledge, enhancing system relevance and accuracy.

The choice between fine-tuning and in-context solutions is closely tied to selecting an encoder or decoder architecture for NLU. The need for regularly updated tools favors encoder-based solutions. Generative models like ChatGPT, while user-friendly, can quickly become outdated or change unexpectedly, compromising the reproducibility and efficiency of prompting techniques (Törnberg, 2024). Additionally, the opacity of their training data makes these models less reliable in zero-shot scenarios. Ethical and legal considerations further limit the use of proprietary generative LLM APIs with private or confidential data, making them unsuitable for most enterprise environments (Törnberg, 2024). Prompt engineering for full KGE is also impractical due to the structural mismatch between natural language and KGs, complicating the creation of satisfactory, automated prompts for large KGs beyond simple proof-of-concept examples.

Thus, we advocate for adapter-based fine-tuning for efficient KGE solutions. Instead of modifying all LLM weights, this approach adds a small network to the existing encoder architecture and trains that module. Adapters are trained on task-specific data, while the original model's weights remain mostly unchanged, acting as prior knowledge. This method is more computationally efficient, allowing LLMs to be plug-and-play components in the data pipeline, making the system more flexible and easily updated. This approach would manage the carbon footprint of extensive computational processes and extend the lifespan of KGE solutions.

### 4.2 Data quality and solution benchmarking

As mentioned, all supervised methods for KGE require creating large, annotated datasets. While leveraging benchmark datasets from literature would be ideal, most of these datasets are built from collaborative KGs. This can pose challenges and performance depletion when trying to export solutions to sparser KGs for NLUs. Additionally, the quality and properties of annotated corpora are significant concerns as manual annotation, the most reliable source, is scarcely available.

According to Bassignana and Plank (2022), cross-dataset and cross-domain setups for RE are particularly deficient. To combat data sparsity, several semi-automatically labeled datasets have been constructed, but they have issues, such as missing relation labels (NA). For example, the NYT10d dataset has 53% incorrect labels, while NYT10m and Wiki20m have 96 and 60% of triples labeled as “NA” (Gao et al., 2021; Lin et al., 2022). Datasets defined as manually annotated often only include human annotations in the test set.

Also popular NER datasets show limitations, such as the limited number of entity classes. For instance, the CoNLL 2003 dataset contains only four entity types, ACE 2005 has 7, and Ontonotes v5 includes 18 entities (Zhang and Xiao, 2024). This scarcity challenges the extraction of diverse entity types for KGs. This can cause robustness problems leading to poor generalization in out-of-domain scenarios (Ma et al., 2023). Moreover, most NER datasets are not constructed from a KG, failing to capture complex KG structures and relationships, which affects the quality and completeness of extracted entities.

Given these challenges, generating KG-centered datasets via DS appears to be the safest choice for custom KGE solutions. However, DS can introduce errors due to its reliance on assumptions that are not always valid (Riedel et al., 2010), especially when KGs and the corpus do not align closely, leading to hallucinations. Furthermore, DS principles struggle to accommodate the evolving nature of knowledge in free texts, as text annotation is based on a static, pre-existing KG.

### 4.3 Ever evolving knowledge and LLMs

Maintaining and updating NLU solutions must account for the evolving nature of language and knowledge. KGE relying solely on DS may be inadequate, as weak annotations come from existing KG entities and relations, limiting the prediction of new types. Enterprises require precise solutions and cannot rely solely on self-/unsupervised tools, necessitating some level of human curation in KG updating methods.

This need can be addressed using the human-in-the-loop (HITL) paradigm, which integrates human expertise into the modeling process to manage ML models uncertainty (Wu et al., 2022). In NLU, HITL methods iteratively correct or predict text annotations. Typically, this involves starting with a small set of annotated data (human-curated or weakly labeled), selecting challenging samples for the model, having humans annotate these samples, updating the model with the new annotations, and repeating the process.

HITL effectively handles scarce or sparse data for NER (Shen et al., 2017), can address mislabeling (Muthuraman et al., 2021) and enhance different stages of the ML pipeline, such as data processing, model training, and inference (Zhang et al., 2019; Klie et al., 2020; Wu et al., 2022). Moreover, this paradigm was already successfully employed to dynamically curate and expand databases based on subject matter expert feedback (Gentile et al., 2019). Although a comprehensive HITL method for KGE does not yet exist, Qian et al. (2020) provides a promising starting point. The authors focus on disambiguating entity names with various textual variations using non-annotated examples, DS to generate pseudo labels, and active learning to address DL models' data requirements. They rank predictions based on model confidence and involve users in labeling the top and bottom elements. This framework could be extended to simultaneously handle KG entities and relations, engaging with the full KG structure.

### 4.4 Need for adaptive hidden representations

As previously described, KGE subtasks are often modeled as classification problems, which pose several issues.

Modeling NER or RE as classification outcomes forces the model to predict an entity or a relation, leaving little room for uncertainty. This is problematic especially given the risk of conceptual hallucinations in LLMs leading to false positives (Peng et al., 2022), an undesirable feature in non-transparent models that can compromise disambiguation tasks.

Furthermore, modeling KGE as a classification problem prevents the correct handling of KGs where multiple relations connect two entities. This affects both disambiguation, which must identify the correct triple in a sentence, and link prediction, which aims to detect the appropriate relation.

KGs are dynamic, frequently updating entities and relationships. While HITL can address this, systems must incorporate new classes or modify existing ones. Classification tasks constrain outcomes by a fixed structure, preventing real-time adaptation to evolving KGs and necessitating full retraining when new relations or entity classes are added, making the process inefficient.

We advocate for designing ML models for downstream tasks to consider KG structures. Instead of classification, representation learning methods should be used to minimize noise impact and manage uncertainty and evolving output structures. Methods like contrastive learning can mimic and learn the principles of symbolic KGs and disambiguation systems, leading to a consistent and dynamic deep-learning approach to KGE.

### 4.5 Outlining the process: a simplified pipeline for expanding knowledge graph relations

**Figure 1:**
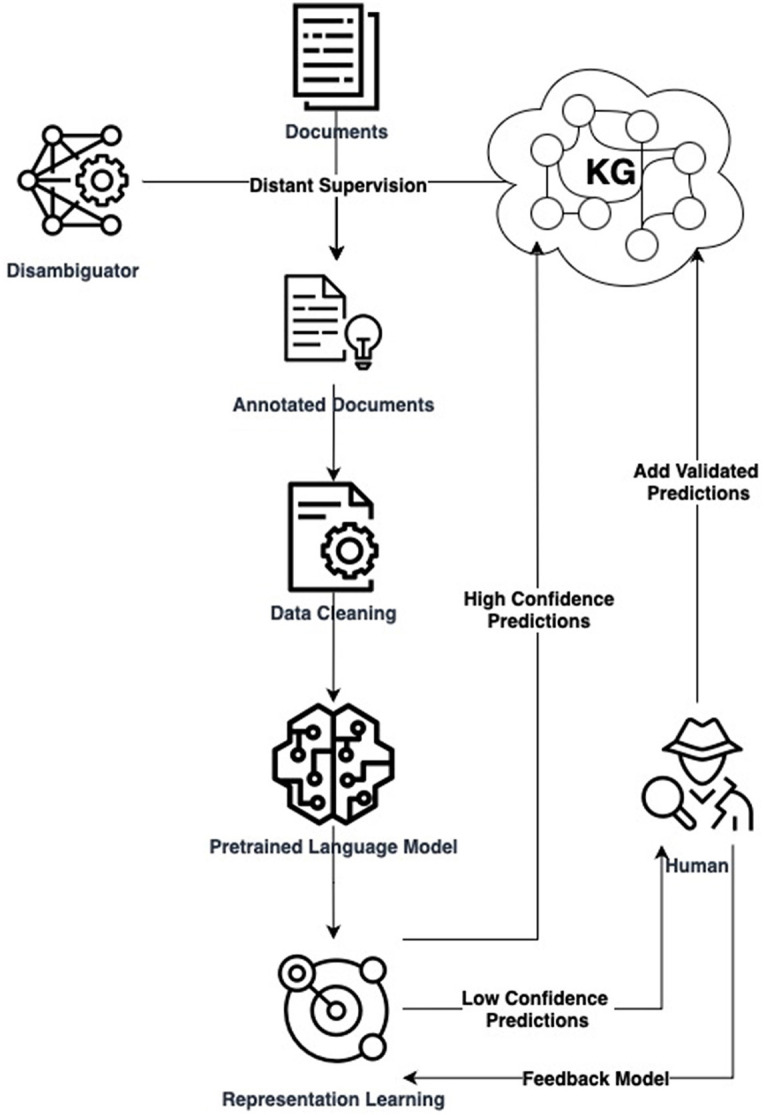
Flowchart illustrates the integration of human feedback in the Expert.AI process, from dataset preparation and disambiguation to knowledge graph querying, data processing, and representation learning.

**Figure 2:**
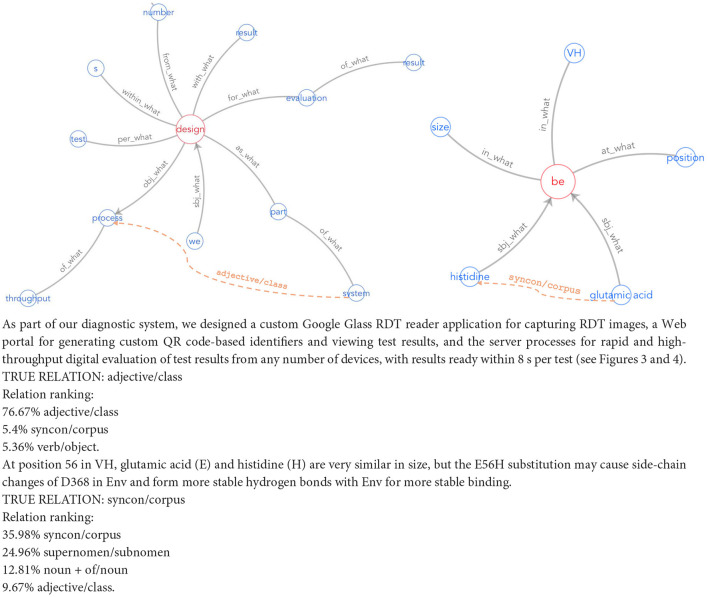
Sample of predicted links in a text. Results of Expert.ai Visualization Tool for RE, represented as solid lines. They show the relations expressing the semantic role of the terms (blue) that are connected to a verb (red). The LLM-based RE framework predicts the relations (ranked in confidence order) shown in the text boxes and the most probable relations are depicted in the corresponding Sensigrafo portions as dashed lines.

Our proposed KGE solution for enterprises involves creating customized datasets via DS, using lightweight supervised representation learning, and integrating human feedback for high-quality updates. Figure 1 illustrates this operational pipeline. Such a pipeline aligns with explainable AI in NLU, addressing computational efficiency, data quality, evolving knowledge, and adaptive representations simultaneously. To illustrate these steps, consider the task of enriching the life sciences-oriented Sensigrafo through RE. We use a collection of PubMed^2^ documents. Entities in the text are marked leveraging Cogito, the Expert.ai's disambiguator, and a DS module grounded on Sensigrafo produces the possible relations in the annotated documents. We select a field-specific PLM, such as BioBERT (Lee et al., 2020). Annotated documents are transformed into contextualized embeddings using the PLM as a prior knowledge base. A small neural network added to the PLM performs adapter-based fine-tuning for RE using techniques like contrastive learning. After training, the model can recognize relations between marked entities in free text, with predictions ranked by model confidence. Figure 2 shows two RE use cases in text boxes. High-confidence predictions are injected into the KG, while low-confidence ones are reviewed by domain experts. Experts validate model results, insert new relations into the KG, and provide feedback by adding new data to the training set. They also assess data quality and identify potential disambiguation mistakes.

## 5 Conclusions

Integrating LLM solutions into enterprise environments reliant on KGs holds great potential for automated and data-driven maintenance and updates. Drawing on the experience of Expert.AI, a leader in NLU solutions, we identify critical issues in current approaches and outline future challenges. Key aspects to address include data quality, computational resources, the role of human expertise, and choosing the right technique to machine-learn the foundational principles of KG construction. Future efforts should aim to develop resilient frameworks that blend automated and human-involved processes, ensuring business applications of LLMs are effective, efficient, and sustainable.

## Data Availability

The original contributions presented in the study are included in the article/supplementary material, further inquiries can be directed to the corresponding author.

## Footnotes

^1^ https://www.expert.ai/products/expert-ai-platform/knowledge-graph/

^2^ https://pubmed.ncbi.nlm.nih.gov/
